# Supplementary figures and images for: Ecology and larval population dynamics of the primary malaria vector Nyssorhynchus darlingi in a high transmission setting dominated by fish farming in western Amazonian Brazil
Source: PLoS One. 2021 Apr 8;16(4):e0246215. doi: 10.1371/journal.pone.0246215 (PMC8031405; doi:10.1371/journal.pone.0246215)

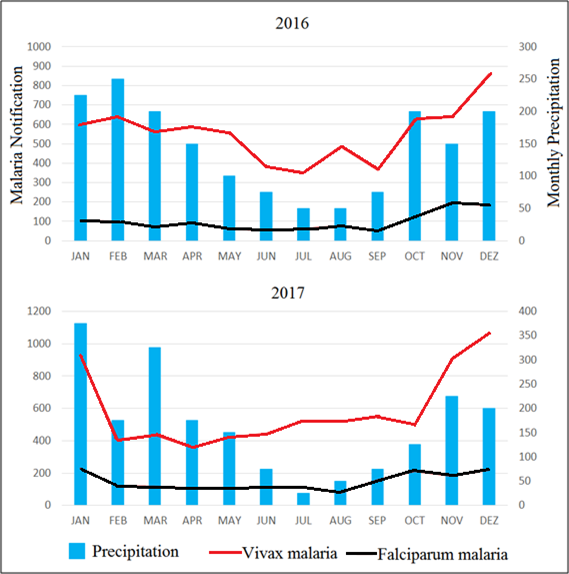

Supplement: S1 Fig — Mancio Lima municipality 2016 and 2017. Pearson correlations: in 2016 for P. vivax and precipitation r = 0.75; for P. falciparum and precipitation r = 0.51; in 2017 for P. vivax and precipitation r = 0.43; for P. falciparum and precipitation r = 0.47. (Source: Malaria: http://www2.datasus.gov.br/DATASUS; Precipitation: clima1.cptec.inpe.br). (TIF) [file pone.0246215.s001.tif]
