# Supplementary material for: Ecology and larval population dynamics of the primary malaria vector Nyssorhynchus darlingi in a high transmission setting dominated by fish farming in western Amazonian Brazil
Source: PLoS One. 2021 Apr 8;16(4):e0246215. doi: 10.1371/journal.pone.0246215 (PMC8031405; doi:10.1371/journal.pone.0246215)
Supplement: Attachment — Submitted filename: Response to Reviewers.docx [file pone.0246215.s005.docx]

**Rebuttal Letter**

To the PLOS One Editors,

Below are our responses to the reviewers of the manuscript we submitted: [PONE-D-21-00873] - [EMID:61ecbed848a59a53], title: Population dynamics of the primary malaria vector *Nyssorhynchus darlingi* in a high transmission setting dominated by fish farming in western Amazonian Brazil.

**1. Please ensure that your manuscript meets PLOS ONE's style requirements, including those for file naming.**

RESPONSE: We modified the text etc. according to the style requirements, including the file names. Minor changes were made in this new version: we chose a new title, to more clearly differentiate this manuscript from previous papers by our group. The Abstract was re-written to in accordance to PLOS recommendations for word count. Author were specified in the “Contributor Roles”, in Authors section on “Manuscript Data” topic in the Editorial Manager.

**2. We note that Figures 1 and 2 in your submission contain map/satellite images which may be copyrighted. All PLOS content is published under the Creative Commons Attribution License (CC BY 4.0), which means that the manuscript, images, and Supporting Information files will be freely available online, and any third party is permitted to access, download, copy, distribute, and use these materials in any way, even commercially, with proper attribution. For these reasons, we cannot publish previously copyrighted maps or satellite images created using proprietary data, such as Google software (Google Maps, Street View, and Earth). For more information, see our copyright guidelines:** [**http://journals.plos.org/plosone/s/licenses-and-copyright**](http://journals.plos.org/plosone/s/licenses-and-copyright)**.**

RESPONSE: We contacted the ESRI company and received permission to use the copyright figures. The new source of figures was the “ArcGIS Online USGS Landsat Baselayer”, and map items were inserted using Microsoft Power Point. The copyright permission request document was provide by ESRI from an owner form (PDF file, attached here), and not the PLOS form model CC BY 4.0, although this permission contemplates full use of these figures, according to the PLOS's requirements about copyright issues.

Yours truly,

Paulo Rufalco-Moutinho

For the authors.
